# The association of *CAPN1* 316 marker genotypes with growth and meat quality traits of steers finished on pasture

**DOI:** 10.1590/S1415-47572009000300011

**Published:** 2009-09-01

**Authors:** María C. Miquel, Edgardo Villarreal, Carlos Mezzadra, Lilia Melucci, Liliana Soria, Pablo Corva, Alejandro Schor

**Affiliations:** Área Genética, Facultad de Ciencias Veterinarias, Universidad de Buenos Aires, Buenos AiresArgentina; 2 Departamento de Producción Animal, Estación Experimental Balcarce, Instituto Nacional de Tecnología Agropecuaria, Balcarce, Buenos AiresArgentina; 3 Departamento de Producción Animal, Facultad de Ciencias Agrarias, Universidad Nacional de Mar del Plata, Balcarce, Buenos AiresArgentina; 4 Laboratorio de Tecnología de Carnes, Facultad de Agronomía, Universidad de Buenos Aires, Buenos AiresArgentina

**Keywords:** beef cattle, tenderness, growth, molecular marker, multivariate analysis

## Abstract

The objective of this paper was to determine the association of a SNP in the μ-calpain gene at position 316 with growth and quality of meat traits of steers grown on pasture. Fifty-nine Brangus and 20 Angus steers were genotyped for *CAPN1* 316. Warner Bratzler shear force was measured in *l. lumborum* samples after a 7-day aging period. A multivariate analysis of variance was performed, including shear force (WBSF), final weight (FW), average daily gain (ADG), backfat thickness (BFT), average monthly fat thickness gain (AMFTG), rib-eye area (REA), and beef rib-eye depth (RED) as dependent variables. The *CAPN1* 316 genotype was statistically significant. Univariate analyses were done with these variables. The marker genotype was statistically significant (p < 0.05) for WBSF (kg: CC: 4.41 ± 0.57; CG: 5.58 ± 0.20; GG: 6.29 ± 0.18), FW (kg: CC: 360.23 ± 14.71; CG: 381.34 ± 5.26; GG: 399.23 ± 4.68), and ADG (kg/d: CC: 0.675 ± 0.046; CG: 0.705 ± 0.016; GG: 0.765 ± 0.014) Shear force, final weight and average daily gain were significantly different according to the *CAPN1* 316 marker genotypes. The marker genotype was statistically significant in the multivariate analysis (p = 0.001). The first characteristic root explained 89% of the differences among genotypes. WBSF, FW and ADG were the most important traits in the first vector, indicating that animals with the marker genotype for lowest WBSF also have the lowest FW and ADG.

## Introduction

Bovine meat quality is defined by several traits that are difficult to evaluate in the live animal. Recently, significant research efforts have focused on the identification of genes influencing production traits in beef cattle, including meat quality. Because of its relevance, meat tenderness has received special attention. In this context, one of the most studied genes is *CAPN1*, which encodes the large subunit of μ-calpain, an enzyme involved in the post-mortem tenderization process (Koohmaraie, 1996). Page *et al.* (2002) were among the first research groups who identified SNPs (Single Nucleotide Polymorphisms) in this gene, which turned out to be associated with differences in tenderness (Page *et al.*, 2004). Soria *et al.* (2006), in a previous work using the same data set as the present paper, confirmed an effect of a SNP in position 316 (*CAPN1* 316) on meat tenderness, measured after a 7-day aging period.

According to Marshall (1999), genetic correlations involving technological quality attributes have not been widely studied, probably due to the difficulties in obtaining appropriate data, although there is some evidence of an association between meat quality and growth traits. When genetic markers are evaluated as possible selection tools for a given trait, it is also necessary to evaluate the consequences on other traits of choosing animals carrying the favorable marker.

The objective of this paper was to determine the association of an SNP in position 316 of the μ-calpain gene with growth and meat quality traits of steers grown on pasture.

## Materials and Methods

A study was conducted on 59 Brangus and 20 Angus steers.

The Brangus steers were representative of the breeds of three different commercial herds (BR1, BR2 and BR3). Each breeder contributed with 20 steers, but one BR3 steer had to be excluded from the analysis because, as its data were out of range (widely exceeding 3σ), it was assumed that an error had occurred in the WBSF measurement. The animals were raised on pastures in their original herds until weaning, which occurred in March, 2004. In April, they were transferred to the Balcarce Experimental Station, National Institute of Agricultural Research (INTA), where they were finished on perennial pastures. Individual sire identifications were not available.

The Angus steers were chosen at random from two herds at the Balcarce Experimental Station. One of these herds produced their own replacements (A1). The cows of the other herd had their origin in this same herd, but commercial bulls had been introduced (A2). Ten A1 and ten A2 steers were used in this experiment. In that particular year (2004), the A1 and A2 herds had been sired by four and seven bulls, respectively. Thus, the animals were grouped in five breed-herd-of-origin groups (A1, A2, BR1, BR2, and BR3).

Fattening started in April, 2004, on perennial, fertilized pastures, when the animals were about 8 to 10 months old, and ended in June, 2005. The steers were weighed monthly, and ultrasound backfat thickness, as well as rib-eye area and depth (smallest diameter) were measured. The steers were slaughtered when at least 50% of a breed-herd group reached 6 mm of backfat thickness. Hence, slaughter took place between March and May, 2005, and the slaughter group was deliberately confounded with the breed-herd group.

The animals were slaughtered at a commercial beef processing facility after 24 h rest in paddocks with available water, following SENASA (National Service for Animal Health) rules. Meat tenderness was measured as Warner-Bratzler shear force (kg) at 7 days post-mortem. After slaughter and following a 24 h cooling period at 1 to 5 °C, the block of steaks corresponding to the 11^th^, 12^th^ and 13^th^ ribs was removed from each left half carcass. The block was deboned and divided into three pieces that were vacuum-packed.

One of these pieces was randomly assigned to maturation treatment at 1-5 °C. After ageing, the meat samples were frozen and kept at -20 °C until they were thawed for the Warner-Bratzler determination (WBSF), performed at the Meat Laboratory of the School of Agriculture at the University of Buenos Aires. Steaks (2.5 cm thick) were thawed at room temperature for 24 h. External fat, peripheral connective tissue and muscles were removed from each steak, leaving only the *longissimus lumborum* muscle. Samples were immersed in plastic bags and boiled in a water bath at 70 °C for 50 min. The cooked steaks were cooled under running tap water for 40 min. The bags were drained and the cuts were gently mopped dry with a paper towel. Five 2.5 cm-diameter cores were removed from each steak, parallel to the muscle fibers. The cores were sheared at their middle point with a 50 kg compression load cell and a Warner-Bratzler V-notch blade mounted on an Instron Testing Machine (model 4442), at a crosshead speed of 50 mm/min. A single peak-shear force measurement was obtained for each core, and these results were averaged to obtain a single WBSF (kg) value for each sample.

###  Genotyping

DNA was extracted from 500 μL of blood using the phenol/chloroform method and ethanol precipitation, and resuspended in 10 mM Tris HCl buffer.

*CAPN1* 316 is a cytosine/guanine (C/G) polymorphism in exon 9 of the *CAPN1* gene on BTA 29 (Page *et al.*, 2002). SNP 316 was genotyped by the PCR-RFLP method (Soria *et al.*, 2006). Primers were selected from the *CAPN1* DNA sequence (GenBank accession AF252504). The primer sequences used for genotyping the 316 marker were:

Forward: CCAGGGCCAGATGGTGAA, and reverse: CGTCGGGTGTCAGGTTGC.

The annealing temperature was 62.5 °C, and the amplified DNA was digested with the *Btg*l enzyme (New England Biolabs, Beverly, MA).

Table 1 shows the distribution of steers per breed-herd of origin group and the *CAPN1* 316 genotypes found (Soria *et al.*, 2006).

###  Statistical analyses

Monthly weight and backfat measurements were used to calculate individual average daily gain (ADG) and average monthly backfat gain (AMBG) by regression. Univariate analyses of variance were performed for the following dependent variables: WBSF, AMBG, ADG, and final weight (FW), which was the last weight taken before slaughter, and the last ultrasound measurements of backfat thickness (BFT), rib-eye area (REA) and rib-eye depth (RED). Fixed effects were the breed-herd group (A1, A2, BR1, BR2, BR3) and the *CAPN1* 316 genotype (CC, CG, GG). Contrast analyses were performed to test for non-additive or dominance effects: CG- (CC+GG) for the statistically significant traits.

A multivariate analysis of variance was made, including WBSF, AMBG, ADG, FW, BFT, REA and RED. The fixed effects were the same as in the univariate analyses, *i.e.*, breed-herd group and *CAPN1* 316 genotype.

It was of special interest to test the null hypothesis of no *CAPN1* genotype effect. The alternative was a genotype effect different from zero. For this purpose, we used the Hotelling-Lawley trace test statistics as the multivariate linear hypothesis and Roys greatest characteristic root statistics (Morrison, 1967).

If statistically significant differences are found between genotypes, the inspection of characteristic vectors allows detecting traits with major influence on those differences.

Correlations of the canonical variables with the traits were calculated, in order to allow subjective comparisons among coefficients. Averages of canonical variables for each *CAPN1* 316 genotype were obtained.

Characteristic roots and vectors were obtained from E -1 H, where H is the matrix of sums of squares and products among *CAPN1* genotypes, and E is the matrix of error sum of squares and products. Since the rank of E -1 H is two, the characteristic roots and vectors obtained were two.

Other statistical calculations performed were: 1) product-moment correlations between the animals' canonical variable and each trait measured in the animal, indicating the importance of the traits in the canonical variable and, consequently, in the differences among *CAPN1* genotypes; 2) means of the two canonical variables for each *CAPN1* genotype, obtained by multiplying the vector of the *CAPN1* genotypes least squares means of the dependent variables by the characteristic vector. The SAS program (SAS Institute Inc., 1999) was used for all analyses.

## Results and Discussion

In the univariate analyses, all traits differed (p < 0.05) among breed-herd groups (results not shown), whereas the marker genotype was significant only for WBSF, ADG and FW (p < 0.05). The least squares means for each marker genotype are presented in Table 2. For those traits for which the differences among genotypes were statistically significant, the CC means were not different from those of CG, and the means of both these genotypes were lower than those of GG (p < 0.05). The lack of significant differences between CC and CG may be due to the high standard errors found for CC, resulting from the low number of animals carrying this genotype. The genotype effects on WBSF, ADG and P15 were consistent with additive marker effects, since none of the tests for non-additive effects performed on them was statistically significant (p = 0.52, 0. 63 and 0.86, respectively).

Multivariate analysis (Hotelling-Lawley Test) indicated differences in genotypes and breed-herd groups. Similarly, the Hotelling-Lawley Trace and Roy's greatest characteristic root tests were statistically significant for genotypes (p = 0.0010 and p < 0.0001 respectively). A difference was also found for breed-herd groups (p < 0.0001, both tests).

Characteristic vectors 1 and 2 for genotypes, their characteristic roots and the correlations between canonical variables and the traits are shown in Table 3.

Eighty-nine percent of the differences between genotypes were due mainly to differences in WBSF, FW and ADG. During fattening, steers which were homozygous for allele G at marker position 316 grew faster (higher ADG), were heavier at a similar backfat thickness, and their meat had higher WBSF values than steers with genotypes CG and CC (Table 2). RED and REA, this last one with a negative coefficient in the vector, also contributed to explain the differences among genotypes, but to a lesser degree.

Vector 2, orthogonal to the first one, explained the remaining 11% of the variance. The correlations of RED and REA with the canonical variable were the highest, while the correlations of all the other traits with the canonical variable were low, indicating their small influence on the differences among genotypes explained by this vector.

BFT and AMBG were not important for the differentiation of genotypes in any one of the vectors. This result was expected, once all steers were slaughtered at a similar BFT.

**Figure 1:**
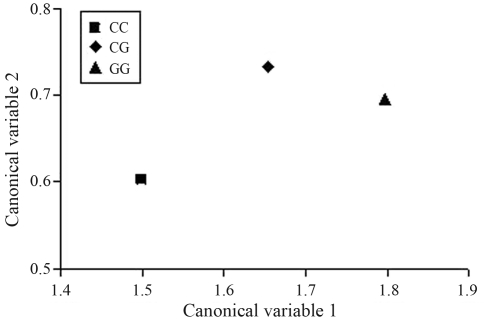
Canonical variables 1 and 2 for the *CAPN1* genotypes which include WBSF: Warner-Bratzler shear force; BFT: backfat thickness; REA: rib-eye area; RED: rib-eye depth; AMBG: average monthly backfat gain; ADG: average daily weight gain; FW: final weight.

The canonical variables 1 and 2 for each genotype are shown in Figure 1. The canonical variable 1 for CG was intermediate between CC and GG, which is in accordance with the fact that the CG least square means were also intermediate between the homozygotes (Table 2) for the important traits in the vector. The canonical variable 2 for CG was higher than those of both homozygotes. This higher value is a consequence of the similar average of genotype CG and GG for RED, and the higher (although not statistically significant) mean value of CG for REA as compared to both homozygotes (Table 2).

According to the results of the multivariate analysis of variance, if animals were selected based on this marker, a genetic improvement regarding WBSF would be expected, as reported by Page *et al.* (2004). But, according to these results, the animals would also be selected for phenotypic traits other than WBSF, such as changes in weight, with constant backfat thickness and daily gain in the fattening finishing period. Moreover, as shown by the characteristic vector 2, changes in rib-eye area and rib-eye depth might be expected, but to a lesser degree.

In the present paper, the analysis was based on phenotypic data, but reports from the literature indicate that, along with loci affecting beef tenderness, other loci associated with weaning weight and carcass weight were mapped to the distal region of bovine chromosome 29 BTA29 (Cattle Quantitative Trait Locus data base). Casas *et al.* (2003) found that chromosome 29 might harbor QTLs for weaning weight, hot carcass weight and WBSF. Support intervals indicate that they are very close and probably overlap. The positions of these QTLs may lead to genetic correlations among these traits.

On the other hand, it is well known that the genetic and phenotypic correlations among weights at different ages are positive (Woldehawariat *et al.*, 1977), as are the correlations (genetic and phenotypic) with carcass weight (Wilson *et al.*, 1976). Thus, it is possible that WBSF shows an association with FW, and that selection for this marker may lead to changes in both traits and also in ADG [important in the vector and correlated genetically with FW (Woldehawariat *et al.*, 1977)]. Thus, if a positive correlation between FW and WBSF is confirmed, selection for FW may result in animals with less tender meat if the animals are selected for this gene.

The importance of REA in canonical variable 1 may be explained by the association of REA with different weights: a positive genetic correlation with carcass weight was reported (Marshall, 1999), which is genetically correlated with slaughter weight (Wilson *et al.*, 1976). It is probable that RED, a lineal measure of the beef, is associated with REA.

The importance of REA and RED in vector 2 being orthogonal to vector 1 is that it indicates that, besides their contribution to the differences among genotypes because of their association with weight, there are other causes contributing to these differences. Although the genotype effect for REA was not statistically significant (Table 2), its genotype means rank was different from the genotype ranks of other traits, with a heterozygote value higher than that of both homozygotes. The RED means also followed a different pattern than those of the other traits, since the means of CG and GG were similar and both were higher than that of CC. This differential behavior of REA and RED with respect to the other analyzed traits is probably enhanced by this vector. The lack of statistical significance in the univariate tests and the low (11%) percentage of the variance explained by this vector are indicative of a weak association between these traits and *CAPN1* 316.

If animals are selected for higher growth, their meat will be tougher, according to vector 1, and intermediate for muscular development, according to vector 2, although, since this vector explained only 11% of the differences among genotypes, probably not much change in muscularity should be expected.

Partial correlation coefficients were obtained among the dependent variables (Table 4) from the error sums of square matrices of the multivariate analysis of variance. The partial correlation between WBSF and FW was negative (Table 4). On the other hand, it had been observed that the coefficients for both traits in the characteristic vectors were positive. Therefore, the association of WBSF and FW appeared to be positive when their averages were analyzed by genotype, but negative when the data were analyzed within breed-herd group and genotype (Table 4). These results suggest that there is a positive phenotypic correlation between WBSF and FW among *CAPN1* genotypes. Therefore, selecting animals for this marker would generate same-sign phenotypic selection for WBSF, FW and ADG, but, in addition to this association, an association among other gene effects and environmental conditions may generate an opposite-sign association. The values of genetic correlations between meat tenderness and growth traits found in the literature are very variable. Marshall (1999) reviewed several papers and concluded that there was a negative genetic correlation of 0.19 with a rank of 0.0 to -0.47, same-sign association as the partial phenotypic correlation found in this paper. However, among the factors that may be causing the variability of the estimates of the genetic correlations between tenderness and other traits, there is the fact that meat tenderness is the result of the action of many genes and pre- and post-slaughter environmental factors, and the experiments required for estimating it are complex and costly. WBSF is highly dependent on the maturation period, so that an average value may not apply to the conditions of this experiment. In the present study, only meat with a seven-day maturation period was taken into account, and the gene considered (*CAPN1*) has an effect on tenderness specifically within this period. No extrapolations can therefore be made to other data sets concerning different meat aging processes.

Genetic correlations are the result that dictate the association of breeding values for genes coding for traits of interest. In this paper, only the phenotypic variability of the traits was analyzed. In view of the results, it would be desirable to generate information that allows working with the genetic variability of the traits studied, which might provide a better explanation for our findings. For example, breeding instead of phenotypic values could be used, which were not available for this data set. A larger number of animals would also be desirable, especially because of the low frequency of genotype CC. It is also possible that in this sample there was a particular association of alleles in chromosome 29, which may not necessarily occur in other samples too. The aging of the meat is a very important factor in determining WBSF values. Therefore, experiments with meat maturing periods different from the 7 days considered here will certainly show different results. Considering one gene at a time, their direct and correlated effects are only a small contribution to the phenotypic result. As the number of individual genes studied increases, it will be possible to disentangle the nature of these complex biological processes taking place in particular environments.

The method of analysis used in this study is suitable for exploring the association of the molecular markers with several traits.

In conclusion, along with tenderness of aged meat, other traits such as final weight and average daily gain differentiated the *CAPN1* 316 marker genotypes in this study. Choosing animals with the favorable *CAPN1* 316 marker genotype for tenderness resulted in selecting animals with lower average daily gain and final weight. Further experiments with larger samples should be conducted, in order to explore the consequences of selection for the marker on other economically important traits. Since antagonism between selection criteria is not uncommon in animal breeding, a careful analysis of correlated responses is required before establishing long-term selection objectives in beef cattle.

## Figures and Tables

**Table 1:** Distribution of steers per breed-herd group and *CAPN1* 316 genotype.

Genotype*	Breed-herd group					Total	Percentage
	A1	A2	BR1	BR2	BR3		
CC	1	1	0	2	0	4	5.0
CG	3	7	9	6	6	31	39.2
GG	6	2	11	12	13	44	55.7

*C: cytosine; G: guanine. A: Angus; BR: Brangus. The number indicates herd of origin.

**Table 2:** Least squares means and standard errors for WBSF, BF, REA, RED, AMBG, ADG and FW by *CAPN1* genotypes.

Trait	*CAPN1* genotype*		
	CC n = 4	CG n = 31	GG n = 44
WBSF (kg)	4.41 ± 0.57^a^	5.58 ± 0.20^a^	6.29 ± 0.18^b^
BFT (mm)	5.86 ± 0.54	6.04 ± 0.19	5.98 ± 0.17
REA (cm^2^)	47.13 ± 3.76	51.86 ± 1.34	50.81 ± 1.19
RED (cm)	5.01 ± 0.28	5.64 ± 0.10	5.65 ± 0.09
AMBG (mm/month)	0.345 ± 0.057	0.365 ± 0.020	0.366 ± 0.018
ADG (kg/d)	0.675 ± 0.046^a^	0.705 ± 0.016^a^	0.765 ± 0.014^b^
FW (kg)	360.23 ± 14.71^a^	381.34 ± 5.26^a^	399.23 ± 4.68^b^

*C: cytosine; G: guanine. WBSF: Warner-Bratzler shear force; BFT: backfat thickness; REA: rib-eye area, RED: rib-eye depth; AMBG: average monthly backfat gain; ADG: average daily weight gain; FW: final weight. ^a,^^b^Row means with common superscripts did not differ (p > 0.05) in univariate tests.

**Table 3:** Characteristic vectors and roots for genotypes, and correlations between the canonical variable and the traits.

	Traits							Root (%)
	WBSF	BFT	REA	RED	AMBG	ADG	FW	
Vector 1	0.0825	0.0059	-0.0044	0.0142	-0.4223	0.4733	0.0029	0.55(89)
Correlation	0.63	0.02	0.33	0.45	0	0.68	0.79	

Vector 2	0.0199	-0.0344	0.0195	0.0989	0.0864	-0.0777	-2 x 10^-4^	0.06(11)
Correlation	0.19	0.23	0.59	0.75	0.19	-0.11	0.18	

WBSF: Warner-Bratzler shear force; BFT: backfat thickness; REA: rib-eye area, RED: rib-eye depth; AMBG: average monthly backfat gain; ADG: average daily weight gain; FW: final weight.

**Table 4:** Partial correlation coefficients among dependent variables.

	WBSF	BFT	REA	RED	AMBG	ADG	FW
WBSF	1.0	-0.21	0.02	0.03	-0.22	-0.23*	-0.27*
BFT		1.0	0.14	0.12	0.79*	0.33*	0.46*
REA			1.0	0.63*	0.02	0.19	0.24*
RED				1.0	-0.02	0.17	0.30*
AMBG					1.0	0.50*	0.54*
ADG						1.0	0.71*

WBSF: Warner-Bratzler shear force; BFT: backfat thickness; REA: rib-eye area, RED: rib-eye depth; AMBG: average monthly backfat gain; ADG: average daily weight gain; FW: final weight. *p < 0.05.
